# Crystal structure of 4-(anthracen-9-yl)pyridine

**DOI:** 10.1107/S2056989021004710

**Published:** 2021-05-11

**Authors:** Meng Zhao, Gang Zhang, Jingmiao Zhang, Shan Huang, Xiuxia Liu, Fei Li

**Affiliations:** a Department of Nuclear Medicine, the Second Hospital of Anhui Medical University, Hefei 230601, People’s Republic of China

**Keywords:** crystal structure, anthracene, C—H⋯π inter­actions, π–π stacking inter­actions

## Abstract

The title compound, C_19_H_13_N, which crystallizes in the monoclinic *C*2/*c* space group with one half-mol­ecule in the asymmetric unit, was synthesized by Suzuki–Miyaura cross-coupling reaction of 9-bromo­anthracene with pyridin-4-ylboronic acid and purified by column chromatography on silica gel. Light-yellow crystals of 4-(anthracen-9-yl)-pyridine suitable for X-ray diffraction were collected by the solvent evaporation method. In the crystal, pairs of mol­ecules are connected by inter­molecular C—H⋯π (pyridine) inter­actions [*d*(H7⋯*Cg*) = 2.7391 (2) Å], forming cyclic centrosymmetric dimers, further resulting in an infinite one-dimensional linear chain along the *c*-axis direction. Weak face-to-face π–π stacking inter­actions [*d*(*Cg*⋯*Cg*) = 3.6061 (2) Å] link neighboring lamellar networks into the supra­molecular structure.

## Abstract 2

The title compound, which crystallizes in the monoclinic *C*2/*c* space group with one half-mol­ecule in the asymmetric unit, was synthesized by Suzuki–Miyaura cross-coupling reaction of 9-bromo­anthracen-2-ylium with pyridin-4-ylboronic acid.

## Chemical context   

Anthracene and its derivatives constitute a very famous class of fluoro­phores that have been widely used in the development of functional fluorescent chemosensors because of their intriguing photophysical properties and chemical stability (Martínez-Máñez *et al.*, 2003 ▸). One of the most important steps in the rational mol­ecule design of anthracene-based chemosensors is the judicious combination with functional chemical recognition moieties, which can be used for monitoring and qu­anti­fying of abnormal physiological changes at the subcellular level (Densil *et al.*, 2018 ▸; Mondal *et al.*, 2014 ▸; Anand *et al.*, 2015 ▸; Shree *et al.*, 2019 ▸). It has been found that 9,10-distyrylanthracene derivatives with restricted intra­molecular rotations often lead to aggregation-induced emission characteristics (Lu *et al.*, 2010 ▸). In recent years, there has been an increased effort to combine anthracene derivatives with N- or O-coordinated single ligands and other attractive mixed ligands in order to construct tunable fluorescent ligands (Dey *et al.*, 2016 ▸; Yao *et al.*, 2019 ▸). As part of our studies in this area, we report herein the synthesis and crystal structure of a fluorescent mono­pyridine ligand, C_19_H_13_N.
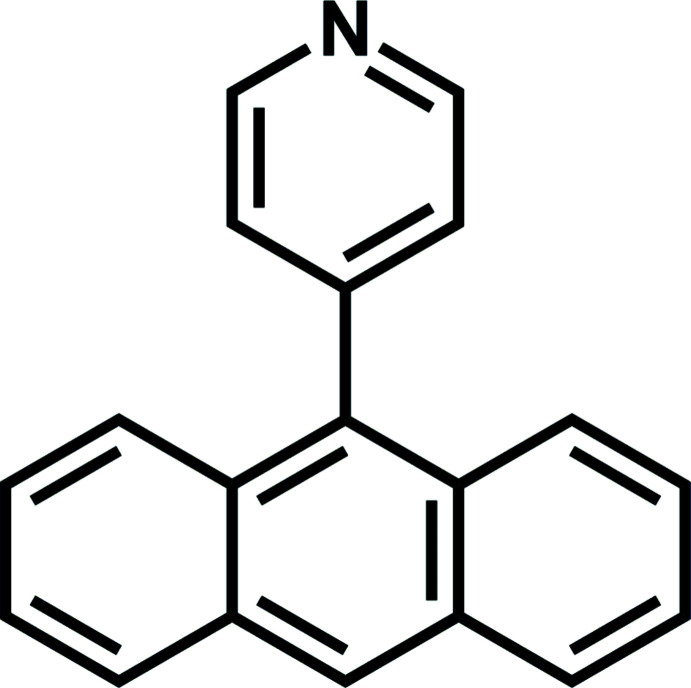


## Structural commentary   

**Figure 1:**
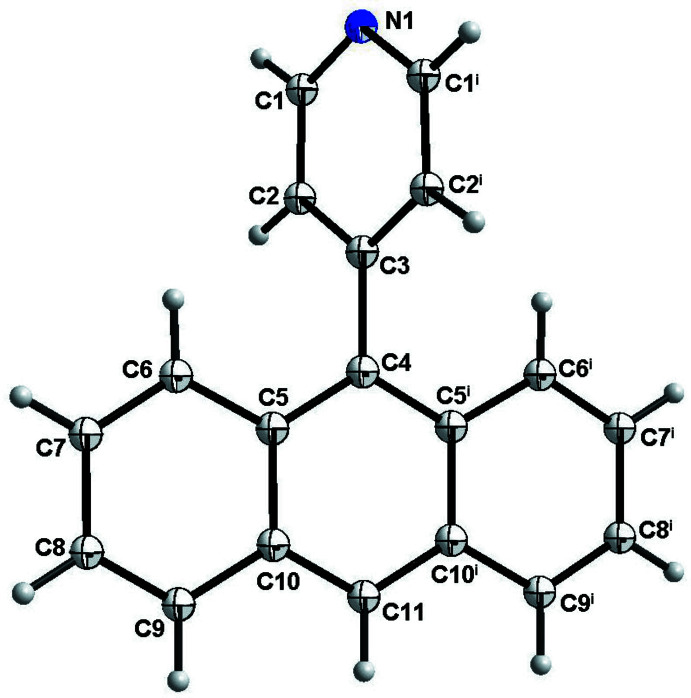
The mol­ecular structure of 4-(anthracen-9-yl)-pyridine with displacement ellipsoids at the 50% probability level. Symmetry code: (i) −*x*, *y*, 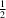 − *z*.

As shown in Fig. 1 ▸, single-crystal X-ray diffraction analysis reveals that 4-(anthracen-9-yl)-pyridine crystallizes in the monoclinic *C*2/*c* space group with half mol­ecule in the asymmetric unit (Table 1 ▸). In the structure of the title compound, the C–C bond lengths of the benzene ring range from 1.3534 (13) to 1.4352 (1) Å, and the C–N bond length is 1.3351 (11), which is comparable with the literature reported (Zhao *et al.*, 2016 ▸). The bond angle of N1–C1–C2 is 124.161 (7)°, closed to the ideal bond angle of 120° for benzene ring. The pyridine ring is inclined to the benzene ring at a dihedral angle of 71.64 (4)°.

## Supra­molecular features   

**Figure 2:**
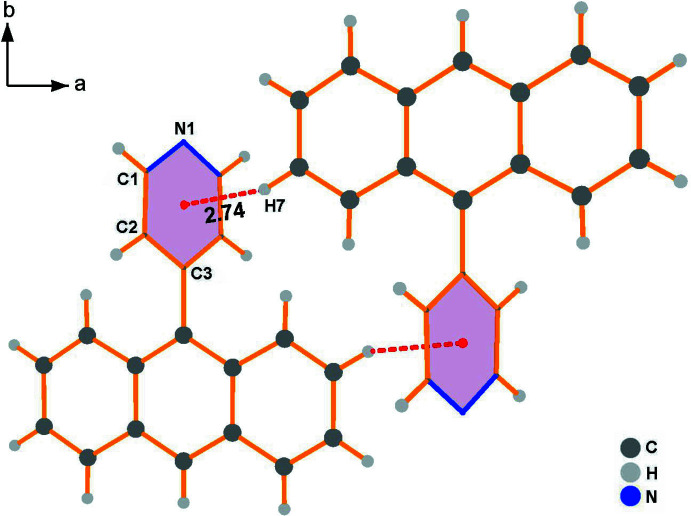
The hydrogen-bonded centrosymmetric dimer along the *c* axis. Dashed lines indicate C—H⋯π inter­actions.

**Figure 3:**
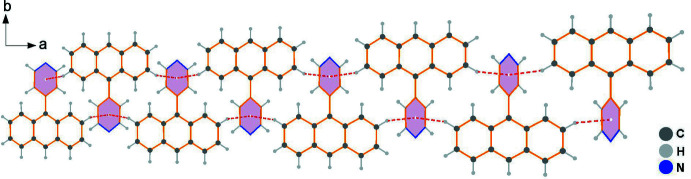
View of the 1-D chain-like structure of the title compound along the *c* axis.

**Figure 4:**
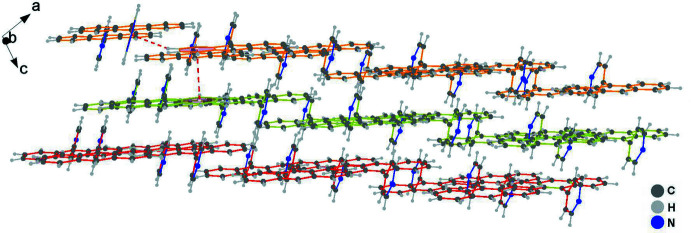
Crystal packing projected *via* C—H⋯π and π–π stacking inter­actions.

In the crystal, the hydrogen atom of anthracene ring contributes to the formation of a C7—H7⋯π contact with the pyridine ring (Table 1 ▸); the resulting cyclic centrosymmetric dimer is shown in Fig. 2 ▸. Subsequently, the paired C—H⋯π(pyridine) hydrogen-bonding inter­actions connect neighboring dimers, resulting in an infinite 1-D linear chain (Fig. 3 ▸), which is basis for extension of the dimensionality. As shown in Figs. 4 ▸ and 5 ▸, the crystal packing involves weak face-to-face π–π stacking inter­actions [*d*(*Cg*⋯*Cg*) = 3.6095 (7) Å] between two benzene rings related by the symmetry operation 1 − *x*, *y*, 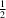 − *z*.

## Database survey   

A search in the Cambridge Structural Database (CSD, Version 5.41, update March 2021; Groom *et al.* 2016 ▸) revealed that this is the first example of a structurally characterized 4-(anthracen-9-yl)-pyridine. At the same time, a CSD search for compounds containing the 4-(anthracen-9-yl)-pyridine substructure identified only one compound, *viz*. Ag_12_(SCH_2_C_6_H_5_)_6_(CF_3_COO)_6_(*L*_4_)_6_ [*L*_4_ = 4-(anthracen-9-yl)-pyridine; Li *et al.*, 2018 ▸] in which the pyridine ring of this compound is inclined to the benzene ring at a dihedral angle of 73.28°.

## Synthesis and crystallization   

**Figure 6:**
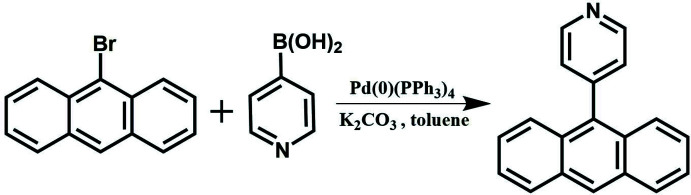
Synthesis of 4-(anthracen-9-yl)-pyridine.

4-(Anthracen-9-yl)-pyridine was synthesized by the Suzuki–Miyaura cross-coupling reaction according to a previously reported protocol (Zhao *et al.*, 2019 ▸). As shown in Fig. 6 ▸, under a nitro­gen atmosphere, 9-bromo­anthracene (2.56 g, 10 mmol), pyridin-4-ylboronic acid (1.23 g, 10 mmol) and tetra­triphenyl phosphine palladium (0.10 g, 0.1 mmol) were dissolved in toluene (90 mL) followed by the addition of potassium carbonate aqueous solution (22 wt%, 40 mL) under constant stirring. The reaction mixture was subsequently refluxed for 12 h, and the mixture was then further purified by column chromatography using petroleum/ethyl acetate (3:1, *v*/*v*) as eluent to give yellow solid 4-(anthracen-9-yl)pyridine (1.76 g, yield 69%).

Crystals of 4-(anthracen-9-yl)-pyridine suitable for X-ray analysis were obtained by the solvent evaporation method. In detail, solid 4-(anthracen-9-yl)-pyridine (0.013 g, 0.05 mmol) was dissolved in 0.5 mL of di­chloro­methane and 5 mL of ethyl acetate. The mixture solvent was evaporated slowly at room temperature for about 2 weeks. Light-yellow crystals of 4-(anthracen-9-yl)-pyridine suitable for X-ray diffraction were collected.

## Refinement   

Crystal data, data collection and structure refinement details are summarized in Table 2 ▸. H atoms were positioned geometrically (C—H = 0.93 Å) and refined as riding with U_iso_(H) = 1.2U_eq_(C).

## Supplementary Material

Crystal structure: contains datablock(s) I. DOI: 10.1107/S2056989021004710/dx2036sup1.cif

Supporting information file. DOI: 10.1107/S2056989021004710/dx2036Isup3.cml

CCDC reference: 2072800

Additional supporting information:  crystallographic information; 3D view; checkCIF report

## Figures and Tables

**Figure 5:**
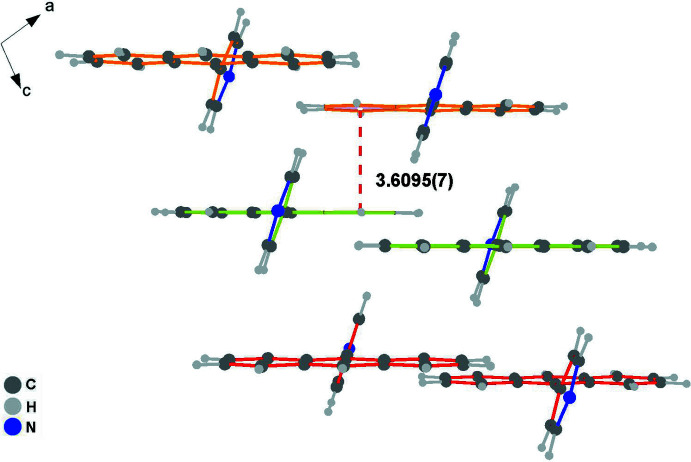
π–π stacking inter­actions of mol­ecules in the crystal structure of the title compound.

**Table 1:** Hydrogen-bond geometry (Å, °) *Cg* is the centroid of the pyridine ring.

*D*—H⋯*A*	*D*—H	H⋯*A*	*D*⋯*A*	*D*—H⋯*A*
C7—H7⋯*Cg* ^i^	0.93	2.74	3.5606 (12)	148
C7—H7⋯*Cg* ^ii^	0.93	2.74	3.5606 (12)	148

Symmetry codes: (i) -x+1, -y+1, -z+1; (ii) x+1, -y+1, z+{\script{1\over 2}}.

**Table 2:** Experimental details

Crystal data	
Chemical formula	C_19_H_13_N
*M*_r_	255.30
Crystal system, space group	Monoclinic, *C*2/*c*
Temperature (K)	296
*a*, *b*, *c* (Å)	6.0777 (4), 20.9211 (16), 10.2574 (7)
β (°)	102.476 (3)
*V* (Å^3^)	1273.45 (16)
*Z*	4
Radiation type	Mo *K*α
μ (mm^−1^)	0.08
Crystal size (mm)	0.18 × 0.15 × 0.13

Data collection	
Diffractometer	Bruker APEXII CCD
Absorption correction	Multi-scan (*SADABS*; Krause *et al.*, 2015 ▸)
*T*_min_, *T*_max_	0.699, 0.745
No. of measured, independent and observed [*I* > 2σ(*I*)] reflections	4703, 1144, 1022
*R*_int_	0.029
(sin θ/λ)_max_ (Å^−1^)	0.602

Refinement	
*R*[*F* ^2^ > 2σ(*F* ^2^)], *wR*(*F* ^2^), *S*	0.036, 0.105, 1.11
No. of reflections	1144
No. of parameters	94
H-atom treatment	H-atom parameters constrained
Δρ_max_, Δρ_min_ (e Å^−3^)	0.15, −0.14

Computer programs: *APEX3* (Bruker, 2018 ▸), *SAINT* (Bruker, 2015 ▸), *SHELXT* (Sheldrick, 2015*a* ▸), *SHELXL* (Sheldrick, 2015*b* ▸) and *OLEX2* (Dolomanov *et al.*, 2009 ▸).

## supplementary crystallographic information

### Crystal data

**Table:**

C_19_H_13_N	*F*(000) = 536
*M**_r_* = 255.30	*D*_x_ = 1.332 Mg m^−^^3^
Monoclinic, *C*2/*c*	Mo *K*α radiation, λ = 0.71073 Å
*a* = 6.0777 (4) Å	Cell parameters from 2979 reflections
*b* = 20.9211 (16) Å	θ = 3.7–25.3°
*c* = 10.2574 (7) Å	µ = 0.08 mm^−^^1^
β = 102.476 (3)°	*T* = 296 K
*V* = 1273.45 (16) Å^3^	Bulk, light yellow
*Z* = 4	0.18 × 0.15 × 0.13 mm

### Data collection

**Table:**

Bruker APEXII CCD diffractometer	1022 reflections with *I* > 2σ(*I*)
φ and ω scans	*R*_int_ = 0.029
Absorption correction: multi-scan (SADABS; Krause *et al.*, 2015)	θ_max_ = 25.3°, θ_min_ = 3.7°
*T*_min_ = 0.699, *T*_max_ = 0.745	*h* = −7→6
4703 measured reflections	*k* = −22→24
1144 independent reflections	*l* = −12→12

### Refinement

**Table:**

Refinement on *F*^2^	0 restraints
Least-squares matrix: full	Hydrogen site location: inferred from neighbouring sites
*R*[*F*^2^ > 2σ(*F*^2^)] = 0.036	H-atom parameters constrained
*wR*(*F*^2^) = 0.105	*w* = 1/[σ^2^(*F*_o_^2^) + (0.0556*P*)^2^ + 0.3319*P*] where *P* = (*F*_o_^2^ + 2*F*_c_^2^)/3
*S* = 1.11	(Δ/σ)_max_ < 0.001
1144 reflections	Δρ_max_ = 0.15 e Å^−^^3^
94 parameters	Δρ_min_ = −0.14 e Å^−^^3^

### Special details

**Table:**

Geometry. All esds (except the esd in the dihedral angle between two l.s. planes) are estimated using the full covariance matrix. The cell esds are taken into account individually in the estimation of esds in distances, angles and torsion angles; correlations between esds in cell parameters are only used when they are defined by crystal symmetry. An approximate (isotropic) treatment of cell esds is used for estimating esds involving l.s. planes.

### Fractional atomic coordinates and isotropic or equivalent isotropic displacement parameters (Å^2^)

**Table:**

	*x*	*y*	*z*	*U*_iso_*/*U*_eq_	
N1	0.000000	0.36240 (5)	0.250000	0.0360 (4)	
C1	0.12815 (18)	0.39626 (5)	0.18480 (11)	0.0359 (3)	
H1	0.219495	0.374032	0.138423	0.043*	
C2	0.13369 (17)	0.46237 (5)	0.18183 (10)	0.0324 (3)	
H2	0.226472	0.483317	0.134422	0.039*	
C3	0.000000	0.49724 (7)	0.250000	0.0266 (4)	
C4	0.000000	0.56870 (6)	0.250000	0.0267 (4)	
C5	0.18833 (15)	0.60220 (5)	0.32231 (9)	0.0279 (3)	
C6	0.38307 (17)	0.57083 (5)	0.39762 (10)	0.0308 (3)	
H6	0.387809	0.526405	0.399226	0.037*	
C7	0.56176 (18)	0.60434 (5)	0.46706 (11)	0.0357 (3)	
H7	0.686262	0.582594	0.515636	0.043*	
C8	0.56061 (19)	0.67201 (5)	0.46633 (12)	0.0405 (3)	
H8	0.683709	0.694491	0.514440	0.049*	
C9	0.38025 (19)	0.70397 (5)	0.39555 (11)	0.0407 (4)	
H9	0.381857	0.748415	0.394805	0.049*	
C10	0.18747 (17)	0.67085 (5)	0.32198 (10)	0.0325 (3)	
C11	0.000000	0.70319 (7)	0.250000	0.0371 (4)	
H11	0.000002	0.747643	0.250003	0.045*	

### Atomic displacement parameters (Å^2^)

**Table:**

	*U*^11^	*U*^22^	*U*^33^	*U*^12^	*U*^13^	*U*^23^
N1	0.0410 (8)	0.0233 (7)	0.0417 (7)	0.000	0.0045 (6)	0.000
C1	0.0393 (6)	0.0281 (6)	0.0414 (6)	0.0039 (4)	0.0110 (5)	−0.0046 (4)
C2	0.0351 (6)	0.0280 (6)	0.0363 (6)	−0.0016 (4)	0.0123 (4)	−0.0006 (4)
C3	0.0274 (7)	0.0243 (7)	0.0269 (7)	0.000	0.0026 (5)	0.000
C4	0.0323 (8)	0.0235 (8)	0.0262 (7)	0.000	0.0108 (5)	0.000
C5	0.0328 (6)	0.0261 (6)	0.0268 (6)	−0.0010 (4)	0.0110 (4)	−0.0001 (3)
C6	0.0351 (6)	0.0260 (6)	0.0320 (6)	−0.0004 (4)	0.0085 (4)	0.0000 (4)
C7	0.0333 (6)	0.0362 (7)	0.0364 (6)	−0.0006 (4)	0.0050 (4)	0.0003 (4)
C8	0.0391 (7)	0.0359 (7)	0.0443 (7)	−0.0109 (5)	0.0039 (5)	−0.0035 (5)
C9	0.0479 (7)	0.0250 (6)	0.0473 (7)	−0.0073 (4)	0.0062 (6)	−0.0017 (4)
C10	0.0393 (7)	0.0255 (6)	0.0333 (6)	−0.0029 (4)	0.0091 (5)	−0.0004 (4)
C11	0.0467 (9)	0.0209 (7)	0.0427 (9)	0.000	0.0075 (7)	0.000

### Geometric parameters (Å, º)

**Table:**

N1—C1	1.3345 (12)	C6—H6	0.9300
N1—C1^i^	1.3345 (12)	C6—C7	1.3579 (15)
C1—H1	0.9300	C7—H7	0.9300
C1—C2	1.3840 (15)	C7—C8	1.4158 (16)
C2—H2	0.9300	C8—H8	0.9300
C2—C3	1.3881 (12)	C8—C9	1.3534 (16)
C3—C4	1.4951 (19)	C9—H9	0.9300
C4—C5^i^	1.4088 (12)	C9—C10	1.4284 (15)
C4—C5	1.4088 (12)	C10—C11	1.3920 (13)
C5—C6	1.4259 (14)	C11—H11	0.9300
C5—C10	1.4362 (16)		
			
C1—N1—C1^i^	115.86 (12)	C7—C6—C5	121.52 (10)
N1—C1—H1	117.9	C7—C6—H6	119.2
N1—C1—C2	124.14 (10)	C6—C7—H7	119.6
C2—C1—H1	117.9	C6—C7—C8	120.73 (10)
C1—C2—H2	120.2	C8—C7—H7	119.6
C1—C2—C3	119.63 (10)	C7—C8—H8	120.0
C3—C2—H2	120.2	C9—C8—C7	119.95 (10)
C2^i^—C3—C2	116.60 (13)	C9—C8—H8	120.0
C2—C3—C4	121.70 (6)	C8—C9—H9	119.3
C2^i^—C3—C4	121.70 (6)	C8—C9—C10	121.38 (11)
C5^i^—C4—C3	119.84 (6)	C10—C9—H9	119.3
C5—C4—C3	119.83 (6)	C9—C10—C5	118.80 (9)
C5—C4—C5^i^	120.33 (13)	C11—C10—C5	119.30 (10)
C4—C5—C6	122.77 (10)	C11—C10—C9	121.90 (11)
C4—C5—C10	119.61 (9)	C10^i^—C11—C10	121.84 (14)
C6—C5—C10	117.62 (9)	C10^i^—C11—H11	119.1
C5—C6—H6	119.2	C10—C11—H11	119.1

Symmetry code: (i) −*x*, *y*, −*z*+1/2.

### Hydrogen-bond geometry (Å, º)

*Cg* is the centroid of the pyridine ring.

**Table:**

*D*—H···*A*	*D*—H	H···*A*	*D*···*A*	*D*—H···*A*
C7—H7···*Cg*^ii^	0.93	2.74	3.5606 (12)	148
C7—H7···*Cg*^iii^	0.93	2.74	3.5606 (12)	148

Symmetry codes: (ii) −*x*+1, −*y*+1, −*z*+1; (iii) *x*+1, −*y*+1, *z*+1/2.
